# Spatial decoupling of light absorption and reaction sites in n-Si photocathodes for solar water splitting

**DOI:** 10.1093/nsr/nwaa293

**Published:** 2020-12-15

**Authors:** Shujie Wang, Tuo Wang, Bin Liu, Huimin Li, Shijia Feng, Jinlong Gong

**Affiliations:** School of Chemical Engineering and Technology, Key Laboratory for Green Chemical Technology of Ministry of Education, Tianjin University, Tianjin 300072, China; Collaborative Innovation Center of Chemical Science and Engineering (Tianjin), Tianjin 300072, China; School of Chemical Engineering and Technology, Key Laboratory for Green Chemical Technology of Ministry of Education, Tianjin University, Tianjin 300072, China; Collaborative Innovation Center of Chemical Science and Engineering (Tianjin), Tianjin 300072, China; School of Chemical Engineering and Technology, Key Laboratory for Green Chemical Technology of Ministry of Education, Tianjin University, Tianjin 300072, China; Collaborative Innovation Center of Chemical Science and Engineering (Tianjin), Tianjin 300072, China; School of Chemical Engineering and Technology, Key Laboratory for Green Chemical Technology of Ministry of Education, Tianjin University, Tianjin 300072, China; Collaborative Innovation Center of Chemical Science and Engineering (Tianjin), Tianjin 300072, China; School of Chemical Engineering and Technology, Key Laboratory for Green Chemical Technology of Ministry of Education, Tianjin University, Tianjin 300072, China; Collaborative Innovation Center of Chemical Science and Engineering (Tianjin), Tianjin 300072, China; School of Chemical Engineering and Technology, Key Laboratory for Green Chemical Technology of Ministry of Education, Tianjin University, Tianjin 300072, China; Collaborative Innovation Center of Chemical Science and Engineering (Tianjin), Tianjin 300072, China; Joint School of National University of Singapore and Tianjin University, International Campus of Tianjin University, Fuzhou 350207, China

**Keywords:** photoelectrochemical (PEC) water splitting, n-Si photocathode, metal-insulator-semiconductor (MIS) junction, light absorption, reaction sites, spatial decoupling

## Abstract

Metal-insulator-semiconductor (MIS) photocathodes offer a simple alternative to p-n junction photocathodes in photoelectrochemical water splitting. However, the parasitic light absorption of catalysts and metal layers in the MIS junction, as well as the lack of low work function metals to form a large band offset with p-Si, severely limit their performance. This paper describes an MIS photocathode fabricated from n-Si, rather than the commonly used p-Si, to spatially decouple light absorption from reaction sites, which enables the majority carriers, instead of the commonly used minority carriers, to drive the surface reaction, making it possible to place the reaction sites far away from the light absorption region. Thus, the catalysts could be moved to the backside of the MIS junction to avoid light shielding. Moreover, the adoption of n-Si unlocks a variety of high work function materials for photovoltage generation. The obtained n-Si MIS photocathode exhibits an applied bias photon-to-current efficiency of 10.26% with a stability up to 300 h.

## Teaser

n-Si was employed to fabricate metal-insulator-semiconductor photocathodes to eliminate the parasitic light absorption and insufficient band offset in traditional p-Si-based photocathodes.

## INTRODUCTION

Photoelectrochemical (PEC) water splitting is a promising approach to converting solar energy into chemical energy in the form of hydrogen [1–3]. Among various photoelectrode materials, crystalline Si (c-Si) has drawn considerable attention because of its narrow bandgap, low cost and mature production technologies. c-Si based metal-insulator-semiconductor (MIS) junctions have been the focus of attention in PEC applications due to their simple fabrication and the potential to achieve higher efficiencies than traditional p-n junctions because of the larger band offset between metal and semiconductor [4]. However, compared with the 11.5% efficiency of n^+^np^+^-Si homojunction photocathode [5], there are very limited MIS Si photocathodes reported with efficiency higher than 5% [6–8]. One of the major challenges for high-efficiency MIS photocathodes is the parasitic light absorption from catalysts and metal layers [4].

Both the catalyst and metal are deposited on the side with light illumination for MIS photocathodes, which leads to decreased light absorption. This is especially true since almost all high performance hydrogen evolution reaction (HER) catalysts (Pt [5], NiMo alloy [9]) are of metallic nature with poor light transmittance. Moreover, the metal layers (Ti, Al [10]) of an MIS photocathode should be thick enough to form a uniform MIS junction, which could worsen the optical loss [11]. Nanosphere lithography-patterning could offer nanoscale-structured metals and catalysts to circumvent the parasitic light absorption partially [6], while the incomplete metal coverage limits the full development of interfacial band bending and effective collection of charge carriers. Therefore, great efforts are needed to avoid parasitic light absorption of the catalyst and metal of MIS.

Another limiting factor for p-Si MIS photocathodes is the lack of appropriate metal with suitable work function ($\Phi$_m_). The band offset between metal and semiconductor, i.e. the difference between the work function of metal and the Fermi-level (*E*_F_) of Si, determines the maximum achievable photovoltage [12]. Unfortunately, Ti ($\Phi$_m_ 4.33 eV) [13] is the only feasibly available low work function metal for p-Si (*E*_F_ ∼ 4.93 eV), which leads to an insufficient band offset (*E*_F_−$\Phi$_m_) of 0.60 eV, resulting in photovoltages lower than 500 mV [6,8]. On the contrary, a wide selection of high work function materials, including nickel ($\Phi$_m_ 5.15 eV) [13], platinum ($\Phi$_m_ 5.65 eV) [13], iridium ($\Phi$_m_ 5.27 eV) [14] and metallic indium tin oxide (ITO, $\Phi$_m_ ∼ 5.05 eV) [15,16], are available for n-Si (*E*_F_ ∼ 4.25 eV) to construct an MIS junction with larger band offset of 0.90, 1.40, 1.02 and 0.80 eV, respectively. Thus, these n-Si MIS junctions are capable of yielding a photovoltage larger than 500 mV [12,15,17,18]. Despite these advantages, n-Si is rarely reported to construct MIS photocathodes due to its limited minority carriers (holes) diffusion length (∼100 μm [19,20]), and thus catalysts must be placed at the same side of the MIS junction to form a photoanode. Additionally, p-Si has been widely chosen to fabricate photocathodes, since p-Si exhibits more suitable conduction band edge position, with respect to the hydrogen evolution reaction, compared to n-Si.

This paper describes the design and fabrication of an illumination-reaction decoupled n-Si MIS photocathode that employs the majority carriers, instead of the minority carriers, to drive the surface reaction. As a result, the MIS junction and catalyst can be placed on the opposite sides of n-Si to avoid the light-shielding problem, breaking through the long-standing bottleneck of parasitic light absorption of catalysts in p-Si MIS photocathodes. Moreover, this MIS photocathode constructed from n-Si instead of p-Si enriches the selection of metallic materials and improves the band offset. Additionally, the adoption of transparent ITO [21] as the high work function metallic material eliminates the parasitic light absorption of metal in MIS while ensuring the uniform formation of the MIS junction. As a result, this illumination-reaction decoupled n-Si MIS photocathode exhibits a light absorption higher than 90% and a photovoltage up to 570 mV.

## RESULTS AND DISCUSSION

### Synthesis of illumination-reaction decoupled n-Si MIS photocathode

**Figure 1.**
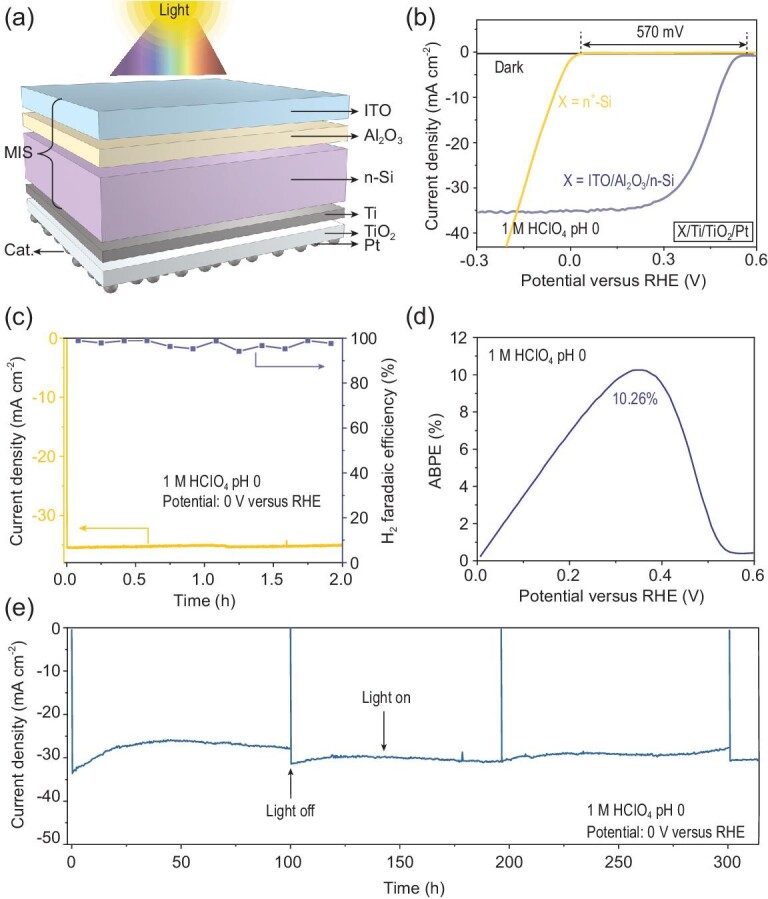
PEC water reduction performance of n-Si MIS photocathode. (a) The schematic of illumination-reaction decoupled n-Si MIS photocathode. (b) *J-V* curves of n-Si MIS photocathode and n^+^-Si/Ti/TiO_2_/Pt cathode. (c) Time-dependent photocurrents (yellow, left-axis) and faradaic efficiency toward H_2_ (purple, right-axis) at 0 V versus RHE for n-Si MIS photocathode. (d) ABPE curves for n-Si MIS photocathode. (e) Stability test of n-Si MIS photocathode at 0 V versus RHE. Data in (b–e) were measured in 1 M HClO_4_ electrolyte (pH 0) under simulated AM 1.5G illumination.

In contrast to traditional p-Si MIS photocathodes with the catalyst deposited on the illuminated side, an original illumination-reaction decoupled n-Si MIS photocathode is designed and fabricated (ITO/Al_2_O_3_/n-Si/Ti/TiO_2_/Pt, Fig. 1a) that is capable of decoupling the light absorption region (n-Si/Al_2_O_3_/ITO MIS junction) from the reaction sites (Pt catalysts), while exhibiting the potential to generate an outstanding photovoltage. For this MIS junction, an optimized 2.5 nm Al_2_O_3_ interlayer was first deposited on the n-Si wafer by atomic layer deposition (ALD) to saturate the dangling bonds on the surface of n-Si/SiO*_x_* as well as suppress the formation of interfacial defects [22,23]. Afterwards, a high work function ITO layer with a thickness of approximately 50 nm and smooth surface (Supplementary Fig. 1a and b) was deposited on Al_2_O_3_ by radio frequency (RF) sputtering to establish a Schottky barrier with n-Si to form the MIS junction. For the catalyst, 2 nm Pt was deposited on the other side of n-Si with respect to ITO [24]. Note that the photo-generated electrons (majority carriers) separated by the MIS junction will transport to the other side of n-Si surface for HER. In order to promote the electron transport at the n-Si/Pt interface as well as prevent Si from corrosion [25,26], a Ti (5.6 nm) layer and an amorphous TiO_2_ (8 nm) layer were inserted between n-Si and Pt by direct-current (DC) sputtering and ALD, respectively. The Ti layer could prevent Si from being oxidized during TiO_2_ deposition to form insulating SiO*_x_* interfacial layer, which is disadvantageous for electron transport [27]. The ohmic contact constructed from n-Si (*E*_F_ ∼ 4.25 eV)/Ti ($\Phi$_m_ 4.33 eV) also promotes electron transport [12]. For the p-Si MIS photocathode, a 2.5 nm thick Al_2_O_3_ tunneling layer, a 5.6 nm thick Ti, an 8 nm thick TiO_2_ protective layer and 2 nm Pt were deposited on the p-Si in sequence under the same conditions as the n-Si MIS photocathodes. The entire fabrication process mainly consists of standard thin film deposition techniques including ALD and RF/DC sputtering, which makes it possible for industrial scaling-up.

### PEC performance of illumination-reaction decoupled n-Si MIS photocathode

To clarify the effectiveness of the illumination-reaction decoupled configuration (Fig. 1a) of this n-Si MIS photocathode for PEC HER, current density-potential (*J-V*) curves were measured in 1.0 M HClO_4_ solution (pH 0) under simulated air mass (AM) 1.5G sunlight illumination. All electrodes show negligible dark current, indicating that they are well sealed without noticeable leakage current. This n-Si MIS photocathode shows an onset potential (defined as the potential required to achieve a cathodic current of 0.1 mA cm^−2^) of 0.57 V versus reversible hydrogen electrode (RHE) and a photocurrent density of ∼35.2 mA cm^−2^ at 0 V versus RHE for PEC HER (Fig. 1b). Upon the adoption of this illumination-reaction decoupled n-Si MIS junction, a photovoltage of 570 mV can be obtained, as evidenced by the difference in onset potential between the n-Si MIS photocathode and the degenerated n^+^-Si cathode (n^+^-Si/Ti/TiO_2_/Pt) that exhibits no photoresponse. The faradaic efficiency (FE) for H_2_ of the n-Si MIS photocathode is close to 100% at 0 V versus RHE under AM 1.5G irradiation for a 2 h period, indicating that almost all the photo-generated electrons are consumed for HER (Fig. 1c). An applied bias photon-to-current efficiency (ABPE) value of 10.26% is obtained at 0.36 V versus RHE (Fig. 1d). The onset potential, photocurrent density at 0 V versus RHE, and ABPE are superior to that obtained from p-Si/Al_2_O_3_/Ti/TiO_2_/Pt photocathode of 0.31 V, 16.1 mA cm^−2^ and 1.68% (Supplementary Fig. 2a and b) and many previously reported conventional p-Si based MIS photocathodes (Supplementary Fig. 3), and even comparable to homogenous p-n Si photocathodes that require high temperature doping and dopant activation (Supplementary Fig. 4 and Supplementary Table 1) [28–30]. Compared with the conventional p-Si MIS photocathodes, the improved PEC performance of this illumination-reaction decoupled photocathode could be attributed to the fact that the catalyst deposited on the non-illuminated side, as well as the large band offset generated from the n-Si based MIS junction.

The stability of the illumination-reaction decoupled photocathodes was evaluated at 0 V versus RHE in 1 M HClO_4_ under AM 1.5G illumination. Although the Ti layer exhibited the potential to be a protective layer [31], the photocurrent showed a fast degradation during the stability test without TiO_2_ (Supplementary Fig. 5). On the contrary, upon the deposition of the TiO_2_ layer, the n-Si MIS photocathode exhibits a robust photocurrent for more than 300 h (Fig. 1e), and the surface morphology (Supplementary Fig. 6a) and *J-V* curves are nearly unchanged after the stability test (Supplementary Fig. 6b), during which the electrolyte was changed every 100 h to ensure the constant test condition. The fluctuation of the photocurrent could be attributed to the accumulation and detachment of H_2_ bubbles on the electrode surface [24]. The stability of this TiO_2_-protected n-Si MIS photocathode outperforms many previous Si-based photocathodes (Supplementary Fig. 7 and Supplementary Table 1). On the other hand, the ITO layer on the other side of this n-Si MIS structure also contributes to the stability of this photocathode. Equally importantly, the ALD-TiO_2_ exhibits negligible impact on *J-V* curves (Supplementary Fig. 8a) and the corresponding ABPE of the photocathode (Supplementary Fig. 8b), because of the suitable conduction band position of TiO_2_ that enables the efficient transfer of electrons between metal Ti and Pt under photocathodic H_2_ evolution conditions [32–34].

### Understanding the decoupling mechanism of n-Si MIS photocathode

**Figure 2.**
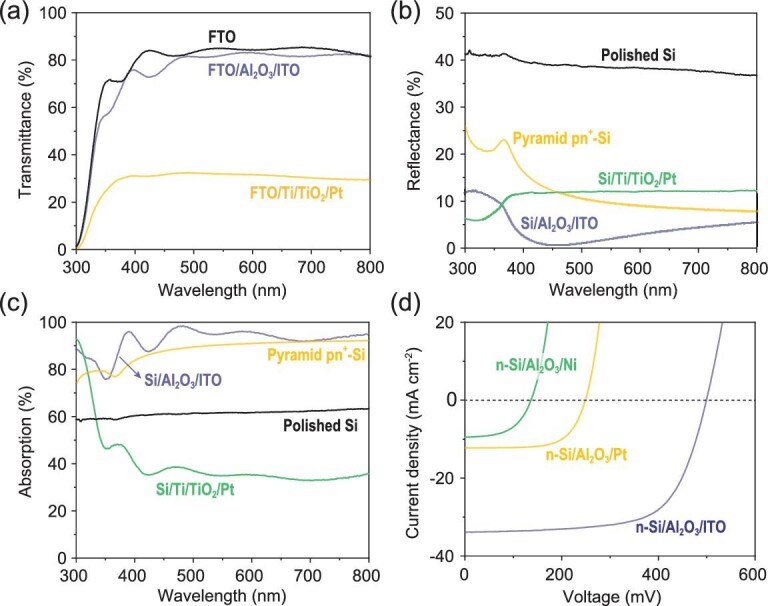
Optical and electrical properties. (a) UV-vis transmission spectra of FTO, FTO/Al_2_O_3_/ITO and FTO/Ti/TiO_2_/Pt. (b) UV-vis reflectance spectra of polished Si, pyramid pn^+^-Si, Si/Al_2_O_3_/ITO and Si/Ti/TiO_2_/Pt. (c) Absorption spectra of polished Si, pyramid pn^+^-Si, Si/Al_2_O_3_/ITO and Si/Ti/TiO_2_/Pt. (d) Solid-state *J-V* characteristics of n-Si/Al_2_O_3_/ITO, n-Si/Al_2_O_3_/Pt and n-Si/Al_2_O_3_/Ni under illumination.

The PEC HER performance of a photocathode would be largely affected by the light absorption of the MIS junction. To quantify the reduction of parasitic absorption from the catalyst realized by this illumination-reaction decoupled configuration, the influence of Pt deposition time on light transmission/reflection was investigated by UV-vis spectroscopy. Fluorine-doped tin oxide (FTO) glass was used as the substrate because the 500 μm Si substrate would completely block the light transmission. When the sputtering duration increases from 5 to 20 s, the light transmittance is approximately reduced by 15%, 28%, 32% and 35%, compared to FTO glass (Supplementary Fig. 9a). Pt sputtering duration shows negligible influence on light reflection when Pt is deposited on bare Si substrates (Supplementary Fig. 9b). According to the light transmittance/reflectance (Supplementary Fig. 9a and b), the parasitic light absorption from 2 nm Pt is about 25% (Supplementary Fig. 9c). Moreover, Ti metal, the only feasibly available metal for a p-Si MIS junction [6,8,10], also reduces the light absorption. The optimal 5.6 nm Ti layer (Supplementary Fig. 10d) deteriorates the loss of light absorption by approximately 35% (Supplementary Fig. 10c), calculated by the light transmission/reflection spectroscopy (Supplementary Fig. 10a and b). For the Al_2_O_3_ and TiO_2_ layers, the light transmission/reflection/absorption remain almost unchanged at their optimal ALD cycles (Supplementary Figs 11 and 12). As a result, only 35% of light can be absorbed by p-Si in p-Si/Al_2_O_3_/Ti/TiO_2_/Pt MIS photocathode in the 400–800 nm region (Fig. 2c), which aggravates the loss of photocurrent.

Upon the formation of this illumination-reaction decoupled n-Si MIS structure, Pt and Ti layers could be moved to the back side of the Si substrate, which decouples the light absorption region from reaction sites. As a result, Pt and Ti layers will not compete with n-Si for light absorption to reduce saturation current density in this unique n-Si MIS photocathode (Supplementary Figs 9d and 10d). Thus, the metallic ITO becomes the only material that determines the parasitic light absorption in this MIS photocathode. According to the UV-vis spectroscopy, the transmittance of FTO/Al_2_O_3_/ITO remains almost unchanged compared with FTO glass (Fig. 2a and Supplementary Fig. 13a). In addition, as an anti-reflective layer, when the ITO film is deposited on the polished Si substrate, the light reflectance is reduced from ∼40% to <5% in the 400–800 nm region, even lower than the pyramidal surface textured (pyramid) pn^+^-Si (Fig. 2b and Supplementary Fig. 13b) [35,36]. Considering the fact that the fabrication of pyramid Si will introduce additional surface charge recombination [37], planer Si is adopted to form the n-Si MIS junction in this study. As a result, the planar n-Si/Al_2_O_3_/ITO MIS photocathode exhibits a maximum light absorption of >90% in the 400–800 nm region, largely surpassing the pyramid pn^+^-Si photocathode and bare polished Si sample (Fig. 2c and Supplementary Fig. 13c), which guarantees a large saturation photocurrent density for the illumination-reaction decoupled n-Si MIS photocathode. The high light transmittance enables the adoption of a thick ITO layer of 46 nm (Supplementary Fig. 13d), which provides sufficient metallic layer coverage to establish a fully developed Schottky barrier for photovoltage generation.

The selection of a high work function metallic layer also promotes the photovoltage generated by the n-Si MIS junction, which will result in a notable difference of PEC HER performance. To compare the influence of different high work function materials on the MIS junction under illumination, solid-state n-Si MIS devices were formed with ITO ($\Phi$_m_ ∼ 5.05 eV, 46 nm), Pt ($\Phi$_m_ 5.65 eV, 10 nm) and Ni ($\Phi$_m_ 5.15 eV, 10 nm) as the metal layers, respectively (schematic illustration in Supplementary Fig. 14). The 10 nm thickness was selected for the two metal layers (Pt and Ni) to ensure that adequate light could transmit into n-Si [4]. The solid-state n-Si/Al_2_O_3_/ITO MIS device shows a higher open-circuit voltage (500 mV) than that obtained from n-Si/Al_2_O_3_/Pt (250 mV) and n-Si/Al_2_O_3_/Ni (137 mV) according to the *J-V* curves (Fig. 2d). The higher open-circuit voltage of the n-Si/Al_2_O_3_/ITO MIS device could be attributed to the more sufficient coverage of the ITO layer than the other two metals as well as the relatively large band offset between n-Si and ITO, which results in more profound band bending. The higher light absorption of the n-Si/Al_2_O_3_/ITO MIS junction than the other two MIS junctions also contributes to the improved photovoltage. Upon the adoption of transparent ITO with a relatively high work function, the trade-off between metal coverage and light absorption confronted by high work function metals is eliminated. Thus, a high photovoltage could be obtained from this n-Si/Al_2_O_3_/ITO MIS junction.

**Figure 3.**
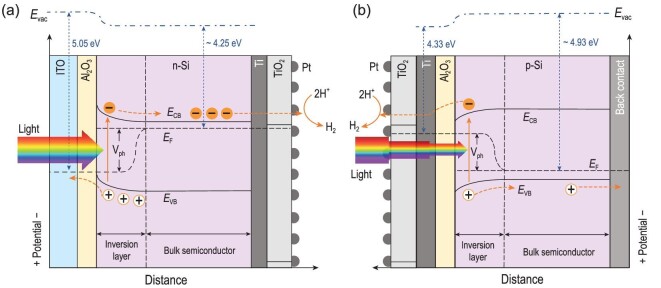
Schematic energy band diagrams. (a) n-Si based photocathode constructed from the n-Si/Al_2_O_3_/ITO MIS junction and (b) p-Si based photocathode constructed from the p-Si/Al_2_O_3_/Ti MIS junction for HER under illumination. *E*_vac_ is the vacuum energy level, *E*_CB_ is the conduction band edge, *E*_VB_ is the valence band edge, *E*_F_ is the Fermi level and *V*_ph_ is the photovoltage generated by the split of quasi-Fermi level under illumination. Layer thickness is not to scale, for clarity purposes.

Based on our experimental results and previous investigations, band diagrams of the n-Si MIS photocathode and p-Si MIS photocathode are illustrated to compare the effectiveness of this illumination-reaction decoupled configuration with the traditional MIS photocathode for light absorption and photovoltage generation during PEC water reduction (Fig. 3). For the illumination-reaction decoupled n-Si MIS photocathode (Fig. 3a), n-Si could adsorb a significant portion of incident light for carrier generation, which is attributed to the adoption of highly transparent and anti-reflective ITO layer as well as the placement of metal Pt and Ti layers to the other side of n-Si. The large photovoltages (*V*_ph_) extracted from the band offset ($\Phi$_m_−*E*_F_, 0.8 eV) between n-Si (*E*_F_ ∼ 4.25 eV) and ITO ($\Phi$_m_ ∼ 5.05 eV) further enhances carrier separation. Therefore, more photo-generated electrons could participate in the hydrogen evolution reaction. For traditional p-Si MIS photocathodes (Fig. 3b), however, only a small part of the light can be absorbed by p-Si because of the serious parasitic light absorption from Pt and Ti, which results in a reduced saturation photocurrent. Moreover, the insufficient band offset (*E*_F_−$\Phi$_m_, 0.6 eV) between p-Si (*E*_F_ ∼ 4.93 eV) and Ti ($\Phi$_m_ ∼ 4.33 eV) results in a smaller photovoltage. Thus, compared with traditional p-Si MIS photocathodes, the adoption of this illumination-reaction decoupled configuration makes it possible to maximize the light absorption of Si light absorber as well as apply the n-Si MIS junction in the photocathode for large photovoltage, which plays a critical role in improving the PEC HER performance.

### Unassisted PEC overall solar water splitting

**Figure 4.**
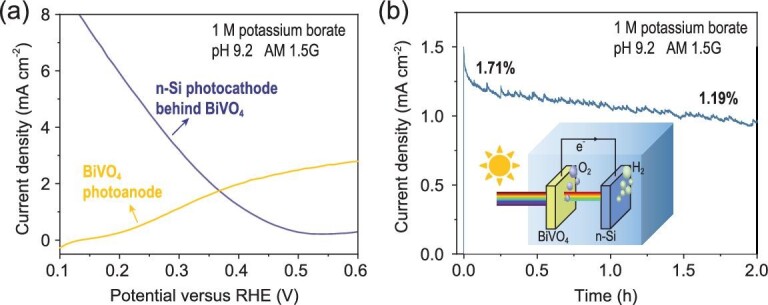
Unbiased water splitting performance of tandem device. (a) *J-V* curves of n-Si MIS photocathode (behind BiVO_4_) and BiVO_4_/FeOOH/NiOOH under simulated AM 1.5G illumination using a three-electrode configuration, respectively. (b) The current-time curve for two-electrode cell with stirring under simulated AM 1.5G illumination. 1 M potassium borate buffer solution (pH 9.2) was used as an electrolyte. Inset: schematic of the BiVO_4_ photoanode and n-Si photocathode tandem water splitting cell.

To illustrate the practical application potential of this illumination-reaction decoupled n-Si MIS photocathode, unassisted overall solar water splitting was demonstrated in a PEC tandem cell (inset of Fig. 4b) composed of this n-Si MIS photocathode and a BiVO_4_/FeOOH/NiOOH photoanode connected in series without external bias, where the light was illuminated from the BiVO_4_ side [38]. A theoretical photocurrent of 1.73 mA cm^−2^ could be expected by the intersection (at 0.37 V versus RHE) of the *J-V* curves of BiVO_4_ and n-Si photocathode (behind BiVO_4_) conducted in the three-electrode configuration, respectively (Fig. 4a). The actual unbiased photocurrent in the two-electrode configuration is 1.39 mA cm^−2^ (Fig. 4b), with a calculated overall solar-to-hydrogen (STH) efficiency of 1.71%. The chronoamperometry test for n-Si MIS photocathode (Supplementary Fig. 15a) shows a stable photocurrent at the operation condition of unbiased tandem cell (0.37 V versus RHE), whereas noticeable photocurrent decay could be observed for the BiVO_4_ photoanode (Supplementary Fig. 15b). Thus, it could be concluded that the decrease of the photocurrent for the tandem cell was caused by the instability of BiVO_4_ in the electrolyte solution [39]. Future study should be focused on improving the stability of BiVO_4_ under operation conditions. Moreover, a monolithic wireless PEC tandem cell may be developed to further improve the feasibility of practical application.

### The versatility of the n-Si MIS junction as photoanode

The ability to eliminate the trade-off between metal coverage and light absorption confronted by high work function metals also makes this n-Si/Al_2_O_3_/ITO MIS junction a versatile photoelectrode for other reactions, such as oxygen evolution reaction (OER). For traditional MIS photoanodes, the metal layer of the MIS junction is the main region that blocks light absorption as most OER catalysts are transparent [40,41]. However, the thickness of the metal layer is limited, which hinders the full development of the Schottky barrier. Upon the adoption of this n-Si/Al_2_O_3_/ITO MIS junction, a high-performance photoanode for PEC water oxidation could be fabricated (n-Si/Al_2_O_3_/ITO/TiO_2_/Ni, Supplementary Fig. 16a). *J-V* characteristics were obtained in 1.0 M KOH (pH 14) under simulated AM 1.5G illumination in three-electrode configuration (Supplementary Fig. 16b). This n-Si MIS photoanode exhibits a large *J*_sc_ of 34.5 mA cm^−2^ as well as a 600 mV photovoltage as indicated by the difference in onset potential (defined as the potential required to achieve an anodic current of 0.1 mA cm^−2^) between the photoanode and the p^+^-Si/Al_2_O_3_/ITO/TiO_2_/Ni with no photoresponse (degenerate p^+^-Si simply acted as a conductive substrate). The ABPE of the photoanode is 1.6% at 1.12 V versus RHE calculated from its *J-V* curve (Supplementary Fig. 16c). The stability under simulated solar illumination was evaluated at an applied potential of 1.65 V versus RHE in 1 M KOH solution [4,32,41], where the photoanode could maintain 85% of its initial photocurrent after photoreaction for more than 60 h (Supplementary Fig. 16d). The *J-V* curves of the photoanode are almost unchanged after the 60 h stability test (Supplementary Fig. 17). The OER performance of this photoanode exceeds most n-Si MIS photoanodes (Supplementary Table 2). Thus, this n-Si MIS junction can be applied to both photocathodes and photoanodes to eliminate the contradiction between light absorption and sufficient energy band bending.

## CONCLUSION

This work designs and fabricates an illumination-reaction decoupled MIS photocathode using n-Si to prevent the parasitic light absorption while establishing a large band offset. Specifically, the majority carriers of n-Si (electrons) are used for hydrogen evolution reaction, so that the light-absorption region and reaction sites can be spatially decoupled to eliminate the light shielding of the catalyst. Moreover, the adoption of n-Si enables a wide selection of metallic materials to form the MIS junction, where ITO is applied to solve the trade-off between light absorption and complete coverage of the metallic layer. Therefore, an enhanced photocurrent density of 35.2 mA cm^−2^ is obtained due to the high transparency and anti-reflection of ITO, while a high photovoltage of 570 mV is extracted from the large band offset between high work function ITO and n-Si. This results in an n-Si MIS photocathode with recorded ABPE value of 10.26%, exceeding traditional p-Si MIS photocathodes. Moreover, an n-Si MIS-BiVO_4_ PEC tandem cell is constructed, achieving a solar to hydrogen conversion efficiency of 1.71% without external bias. The versatility of this n-Si/Al_2_O_3_/ITO MIS junction is demonstrated as a photoanode for OER, which exhibits an ABPE value of 1.6%. This work provides a facile strategy to eliminate the contradiction between light absorption and catalytic activity in p-Si MIS photocathodes, with the potential to further improve PEC performances in other photo-reduction systems that use catalysts with poor light transmittance.

## METHODS

### Materials

Phosphorus-doped, boron-doped, degenerately boron-doped and degenerately arsenic-doped Si wafers were purchased from MTI Corporation. All the sputtering targets were purchased from Zhongnuo Advanced Material (Beijing) Technology Co., Ltd. Trimethyl aluminum (TMA) and titanium (IV) i-propoxide (TTIP) were purchased from Suzhou Fornano Electronics Technology Co., Ltd. All the reagents were used directly without further purification.

### Fabrication of n-Si MIS photocathodes

After cleaning by the piranha solution and HF solution, a 2.5 nm Al_2_O_3_ layer was deposited on the n-Si using a home-built ALD system with TMA and H_2_O as precursors. Afterwards, a 46 nm ITO film was deposited using RF sputtering to form an MIS junction. The MIS junction was heated to 400°C for 30 min in an N_2_ environment. A 5.6 nm Ti film was deposited on the other side of the Si wafer using DC sputtering. Then, an 8 nm TiO_2_ layer was deposited on the Ti film using ALD with TTIP and H_2_O as precursors. Finally, a 2 nm Pt layer was deposited on the TiO_2_ using DC sputtering. After depositions, Cu wire was connected to the ITO film by silver conductive adhesive. The exposed edges and some parts of the front of the electrodes were sealed with an epoxy adhesive.

### Fabrication of n-Si MIS photoanodes

For the photoanodes, a 2.5 nm Al_2_O_3_ tunneling layer, a 20 nm ITO and an 8 nm TiO_2_ protective layer were deposited on the n-Si in sequence under the same conditions as the photocathodes. Then, a 4 nm metallic Ni thin film was deposited on the TiO_2_ film using DC sputtering.

### Fabrication of p-Si MIS photocathodes

For the p-Si MIS photocathodes, a 2.5 nm Al_2_O_3_ tunneling layer, a 5.6 nm Ti, an 8 nm TiO_2_ protective layer and 2 nm Pt were deposited on the p-Si in sequence under the same conditions as the n-Si MIS photocathodes.

### PEC measurements

For photocathodes, the prepared electrode, saturated Ag/AgCl electrode and platinum foil were used as a working electrode, reference electrode and counter electrode, respectively. 1 M Perchloric acid was used as the working electrolyte. For photoanodes, the prepared electrode, Hg/HgO electrode and platinum foil were used as a working electrode, reference electrode and counter electrode, respectively. 1 M potassium hydroxide pellets was used as the working electrolyte. *J-V* curves and chronoamperometry were measured by an electrochemical workstation under the irradiation provided by an AM 1.5G solar simulator.

The ABPE of the electrodes above was calculated from the *J-V* curves, according to the equation ABPE = I × {(1.23 − |V_b_|)/P} × 100% [8]. Where I is the photocurrent density (mA cm^−2^), V_b_ is the potential versus ideal counter electrode (V), and P is the incident illumination intensity (100 mW cm^−2^).

H_2_ was collected and analyzed by an on-line gas chromatograph with a thermal conductivity detector (TCD) using N_2_ as the carrier gas. The FE for the H_2_ product was calculated according to the equation FE (%) = (moles products × number of electrons needed)/(moles of electrons passed) × 100%.

### Characterization

The morphology was characterized using a field emission scanning electron microscope. The thicknesses of the ITO, Al_2_O_3_, TiO_2_, Pt and Ni layers on the polished Si were obtained using a spectroscopic ellipsometer. *J-V* curves of the solid-state cells were measured on a source-meter. The transmission and reflection spectra were recorded by a SHIMADZU UV-2550 spectrophotometer.

## Supplementary Material

nwaa293_Supplemental_File
