# Supplementary material for: Spatial decoupling of light absorption and reaction sites in n-Si photocathodes for solar water splitting
Source: Natl Sci Rev. 2020 Dec 15;8(8):nwaa293. doi: 10.1093/nsr/nwaa293 (PMC8363328; doi:10.1093/nsr/nwaa293)
Supplement: nwaa293_Supplemental_File [file nwaa293_supplemental_file.pdf]

Supporting Information

Spatial Decoupling of Light Absorption and Reaction Sites in n-Si Photocathodes for Solar Water Splitting

Shujie Wang[1,2,#], Tuo Wang[1,2,#], Bin Liu[1,2], Huimin Li[1,2], Shijia Feng[1,2], and Jinlong Gong[1,2,3,*]

[1]*School of Chemical Engineering and Technology; Key Laboratory for Green Chemical Technology of Ministry of Education, Tianjin University; Tianjin 300072, China;*

[2]*Collaborative Innovation Center of Chemical Science and Engineering (Tianjin), Tianjin 300072, China;*

[3]*Joint School of National University of Singapore and Tianjin University, International Campus of Tianjin University, Binhai New City, Fuzhou 350207, China.*

# These authors contributed equally to this work.

* Corresponding author: jlgong@tju.edu.cn. Fax: +86-22-87401818

Supplementary Figures

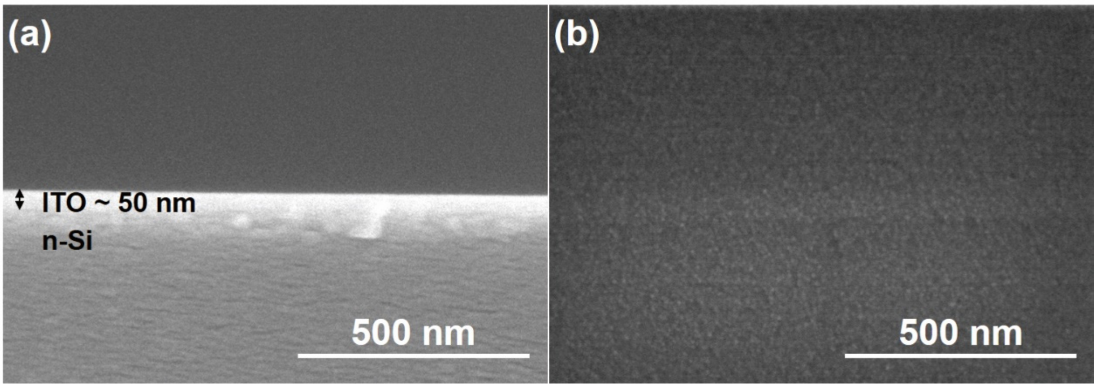

Supplementary Figure 1. Physical characterization of ITO layers. (a) Cross-sectional SEM images and (b) top-view SEM images of n-Si/Al_2O_3/ITO.

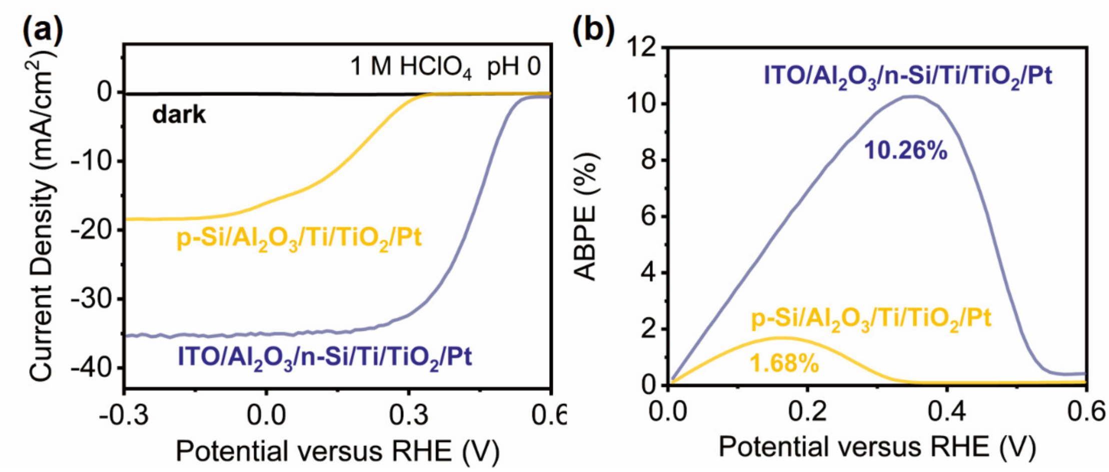

Supplementary Figure 2. PEC water reduction performance comparison of n-Si and p-Si based MIS photocathodes. (a) J-V curves and (b) corresponding ABPE curves of n-Si MIS photocathode and p-Si MIS photocathode.

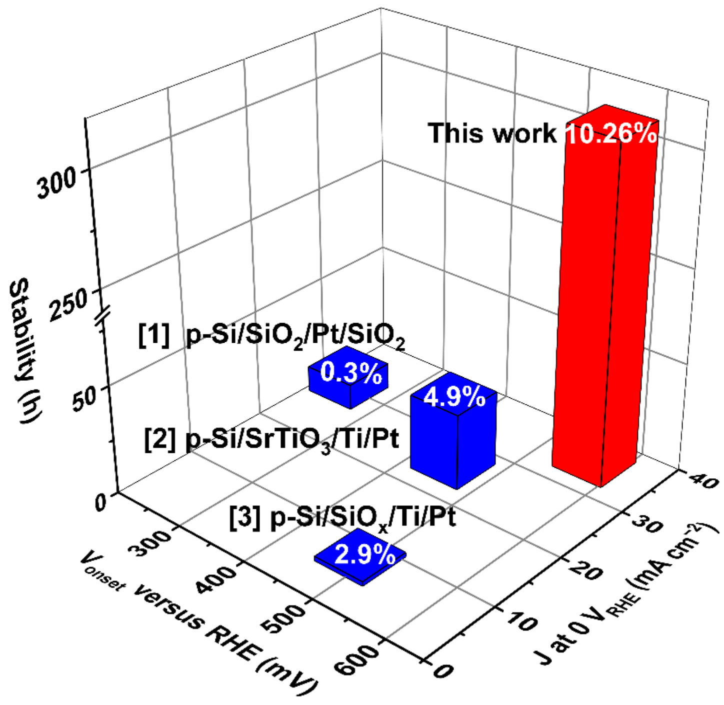

Supplementary Figure 3. PEC water reduction performance comparison of c-Si based MIS photocathodes. Chart visualizing data on reported onset potential value, the current density at 0 V versus RHE and the stability of MIS junction photocathodes for water reduction. Blue bars are referred to the recent reported MIS junction photocathodes and the red bar is referred to this work. The percentage on the bars represents corresponding ABPE values. Details are provided in Table S1.

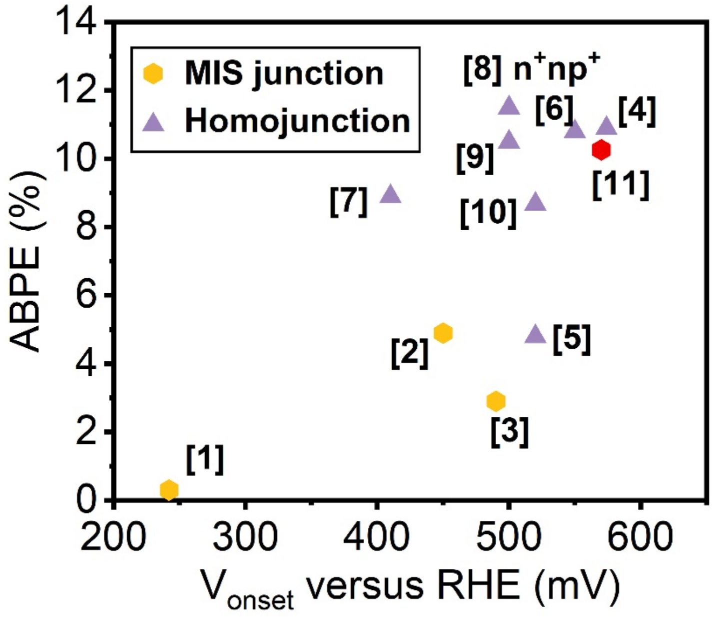

Supplementary Figure 4. Chart visualizing data on reported ABPE value and onset potential value of silicon-based photocathodes for water reduction. Red and yellow hexagons are referred to this work and other MIS junction photocathodes, respectively. Purple triangles are referred to homojunction photocathodes. Details are provided in Table S1.

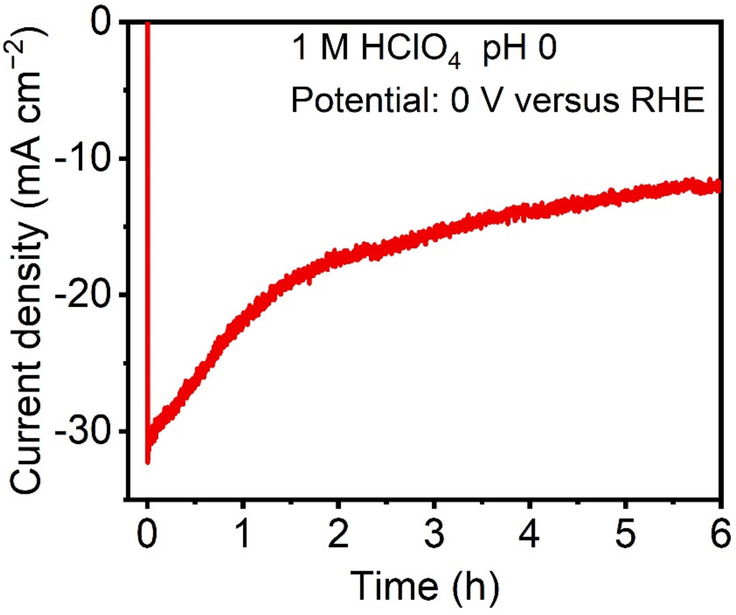

Supplementary Figure 5. Stability evaluation of n-Si MIS/Ti/Pt photocathode. Photocurrent density-time curve of n-Si MIS/Ti/Pt at 0 V versus RHE in 1 M $HClO_4$ electrolyte (pH 0) under simulated AM 1.5G illumination.

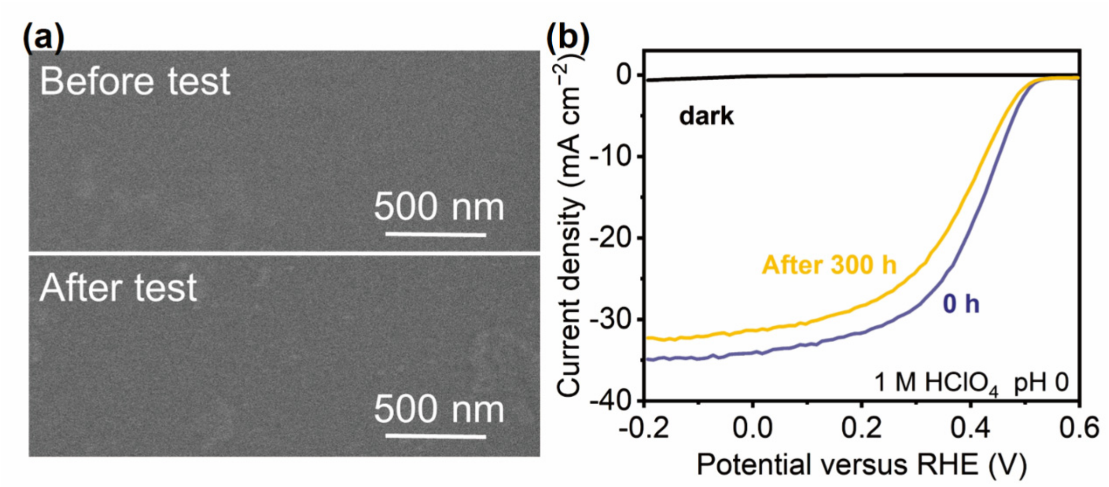

Supplementary Figure 6. Stability evaluation of n-Si MIS photocathode. (a) SEM images and (b) *J*-*V* curves of n-Si MIS photocathodes before and after 300 h stability measurement.

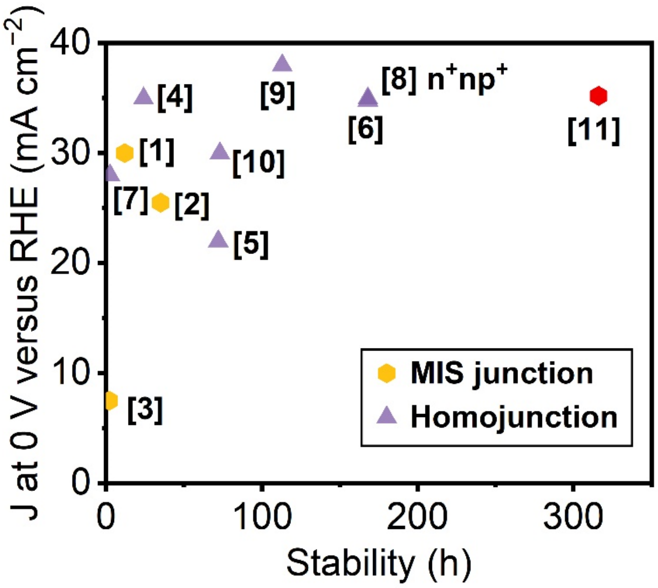

Supplementary Figure 7. Chart visualizing data on reported stability and current density at 0 V versus RHE of silicon-based photocathodes for water reduction. Red and yellow hexagons are referred to this work and other MIS junction photocathodes, respectively. Purple triangles are referred to homojunction photocathodes. Details are provided in Table S1.

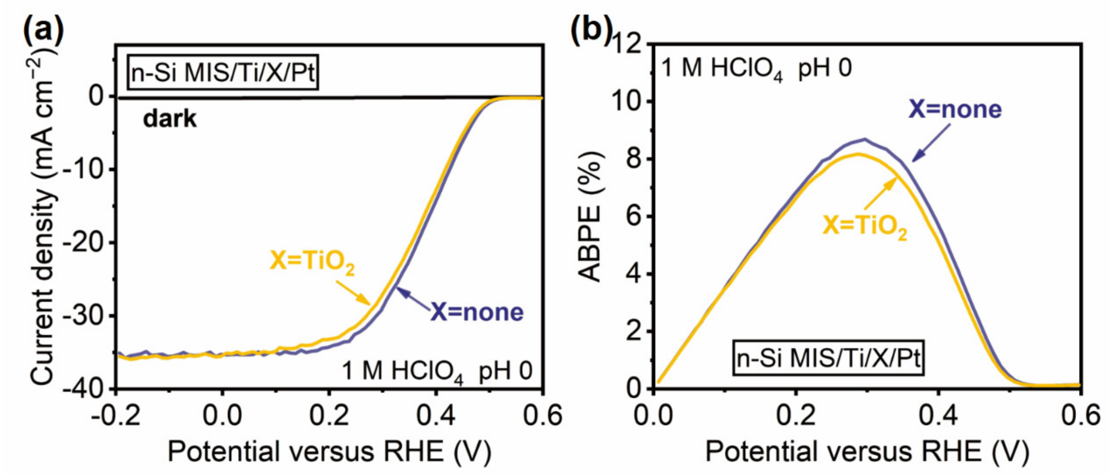

Supplementary Figure 8. PEC water reduction performance of n-Si MIS/Ti/Pt and n-Si MIS/Ti/TiO_2/Pt photocathodes. (a) *J*-*V* curves and (b) corresponding ABPE curves stability test in 1 M $HClO_4$ electrolyte (pH 0) under simulated AM 1.5G illumination.

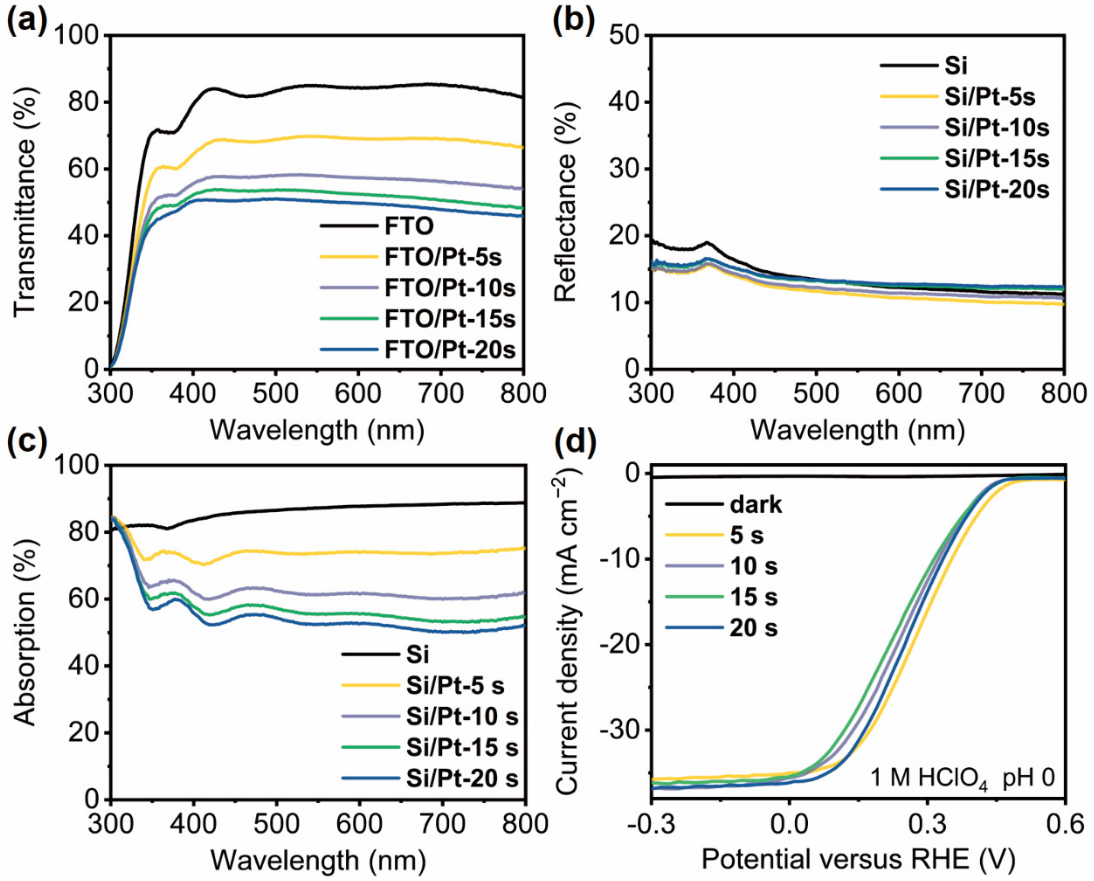

Supplementary Figure 9. Effect of Pt sputtering duration on optical properties and HER performance. (a) UV-vis transmission spectra of FTO/Pt with different Pt sputtering durations. (b) UV-vis reflectance spectra of Si/Pt with different Pt sputtering durations. (c) Absorption spectra of Si/Pt with different Pt sputtering durations. (d) *J-V* curves of n-Si MIS/Ti/TiO_2/Pt photocathodes with different Pt sputtering durations. The thickness of Pt is confirmed by spectroscopic ellipsometry. Pt deposition time of 10 s is used to deposit 2 nm of Pt.

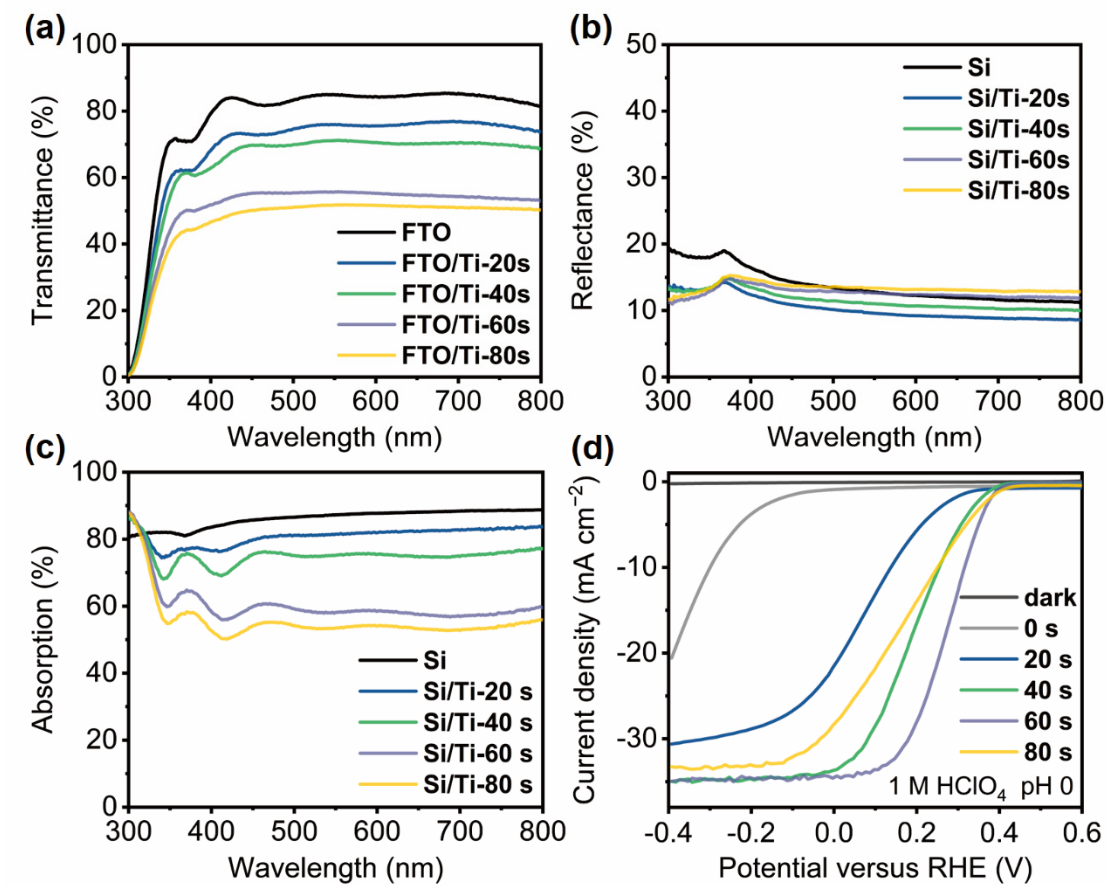

Supplementary Figure 10. Effect of Ti sputtering duration on optical properties and HER performance. (a) UV-vis transmission spectra of FTO/Ti with different Ti sputtering durations. (b) UV-vis reflectance spectra of Si/Ti with different Ti sputtering durations. (c) Absorption spectra of Si/Ti with different Ti sputtering durations. (d) *J-V* curves of n-Si MIS/Ti/TiO_2/Pt photocathodes with different Ti sputtering durations. The thickness of Ti is confirmed by spectroscopic ellipsometry. Ti deposition time of 60 s is used to deposit 5.6 nm of Ti.

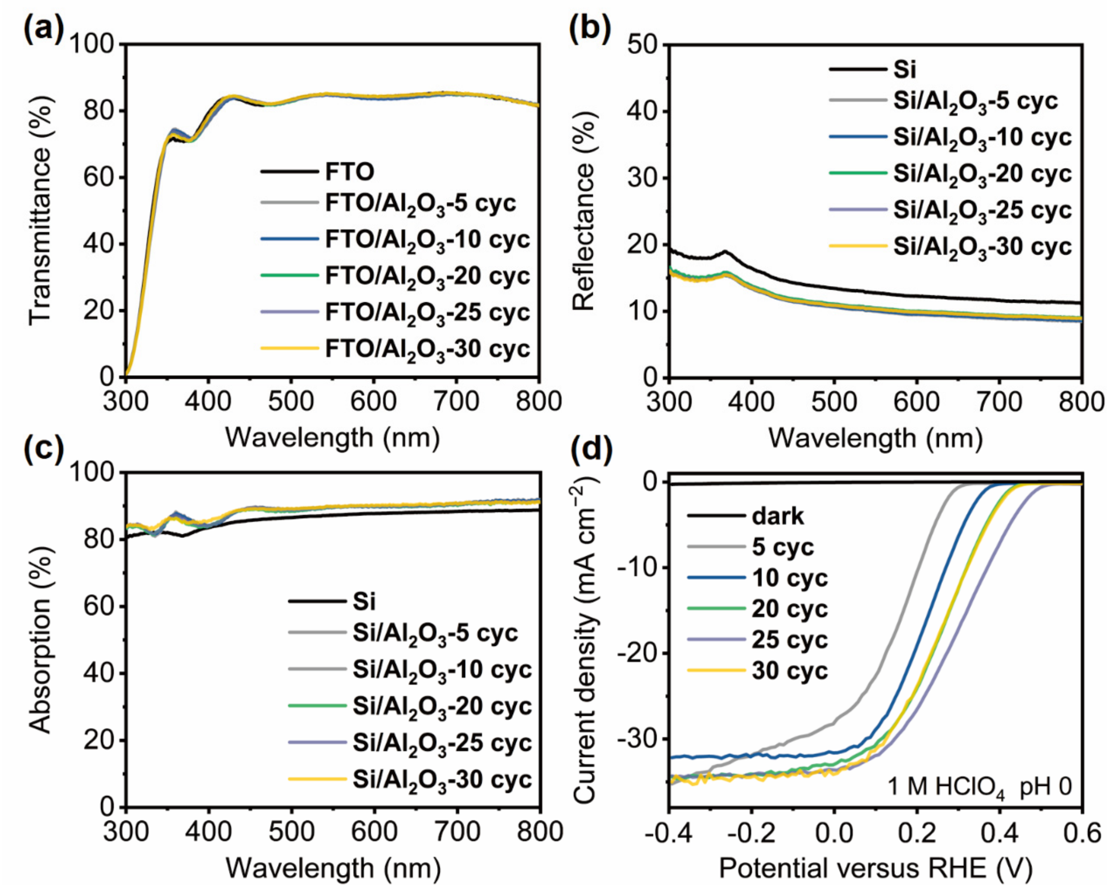

Supplementary Figure 11. Effect of Al_2O_3 deposition cycles on optical properties and HER performance. (a) UV-vis transmission spectra of FTO/Al_2O_3 with different numbers of (TMA + H_2O) ALD cycles. (b) UV-vis reflectance spectra of Si/Al_2O_3 with different numbers of (TMA + H_2O) ALD cycles. (c) Absorption spectra of Si/Al_2O_3 with different numbers of (TMA + H_2O) ALD cycles. (d) *J-V* curves of n-Si MIS/Ti/TiO_2/Pt photocathodes with different numbers of (TMA + H_2O) ALD cycles. The thickness of Al_2O_3 is confirmed by spectroscopic ellipsometry. (TMA + H_2O) ALD cycles of 25 is used to deposit 2.5 nm of Al_2O_3.

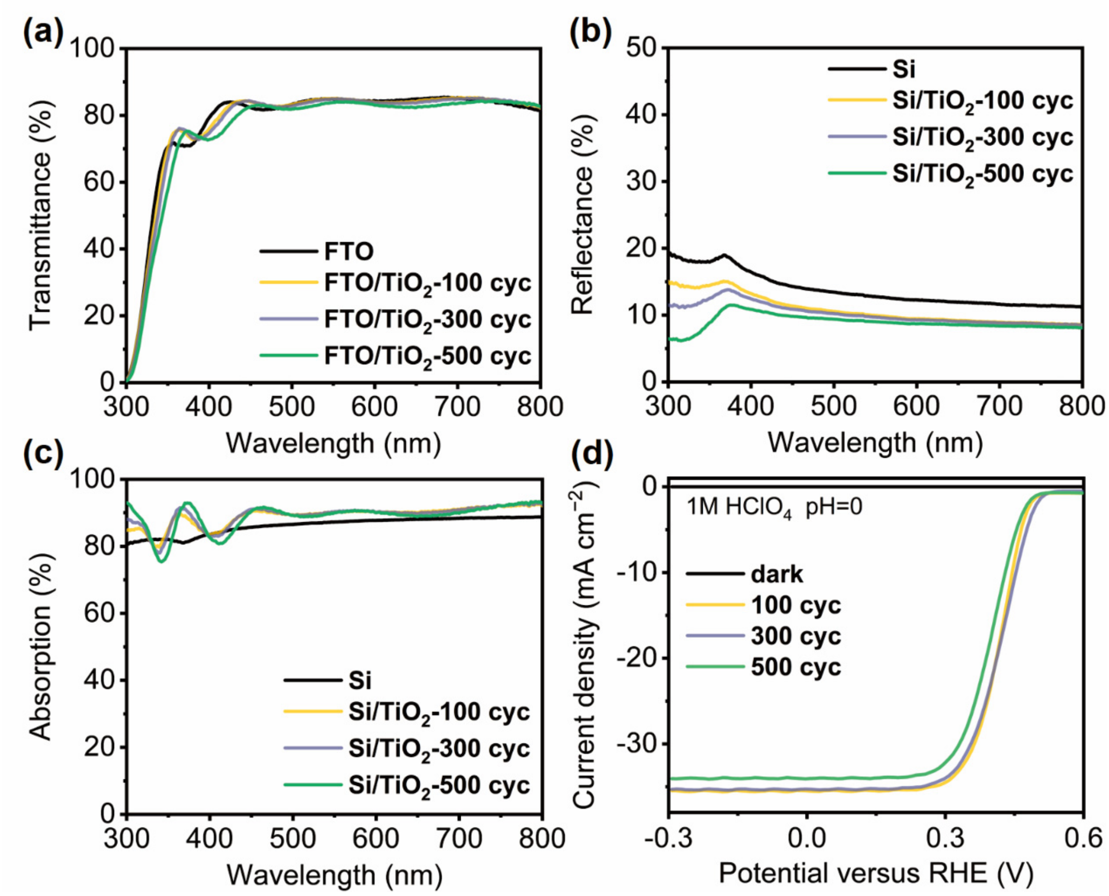

Supplementary Figure 12. Effect of TiO_2 deposition cycles on optical properties and HER performance. (a) UV-vis transmission spectra of FTO/TiO_2 with different numbers of (TTIP + H_2O) ALD cycles. (b) UV-vis reflectance spectra of Si/TiO_2 with different numbers of (TTIP + H_2O) ALD cycles. (c) Absorption spectra of Si/TiO_2 with different numbers of (TTIP + H_2O) ALD cycles. (d) *J-V* curves of n-Si MIS/Ti/TiO_2/Pt photocathodes with different numbers of (TTIP + H_2O) ALD cycles. The thickness of TiO_2 is confirmed by spectroscopic ellipsometry. (TTIP + H_2O) ALD cycles of 300 is used to deposit 8 nm of TiO_2.

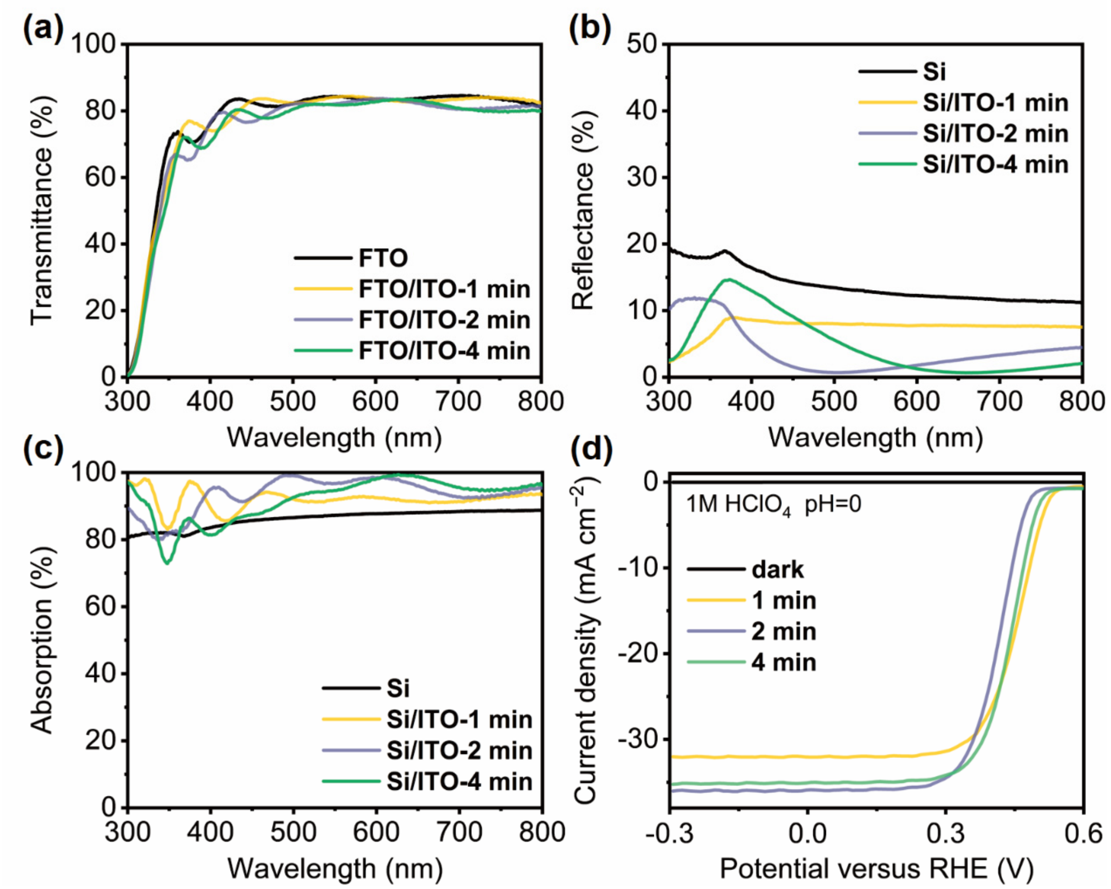

Supplementary Figure 13. Effect of ITO sputtering duration on optical properties and HER performance. (a) UV-vis transmission spectra of FTO/ITO with different ITO sputtering durations. (b) UV-vis reflectance spectra of Si/ITO with different ITO sputtering durations. (c) Absorption spectra of Si/ITO with different ITO sputtering durations. (d) *J*-*V* curves of n-Si MIS/Ti/TiO_2/Pt photocathodes with different ITO sputtering durations. The thickness of ITO is confirmed by spectroscopic ellipsometry and cross-sectional SEM image. ITO sputtering time of 2 min is used to deposit 46 nm of ITO.

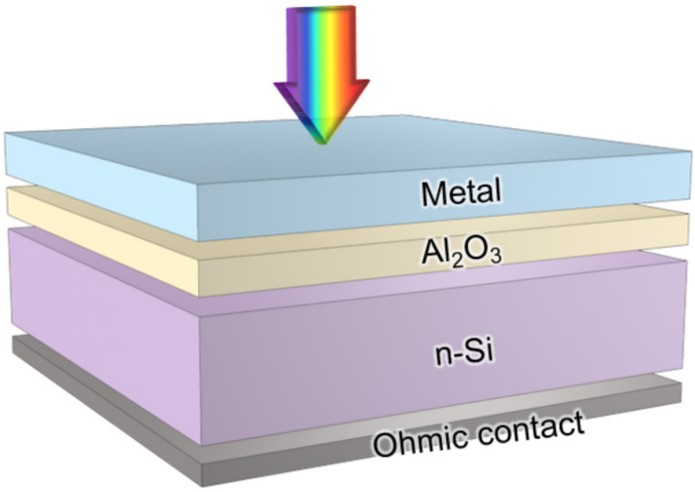

Supplementary Figure 14. Schematic illustration of the solid-state devices of n-Si/Al_2O_3/metal (ITO, Pt, Ni).

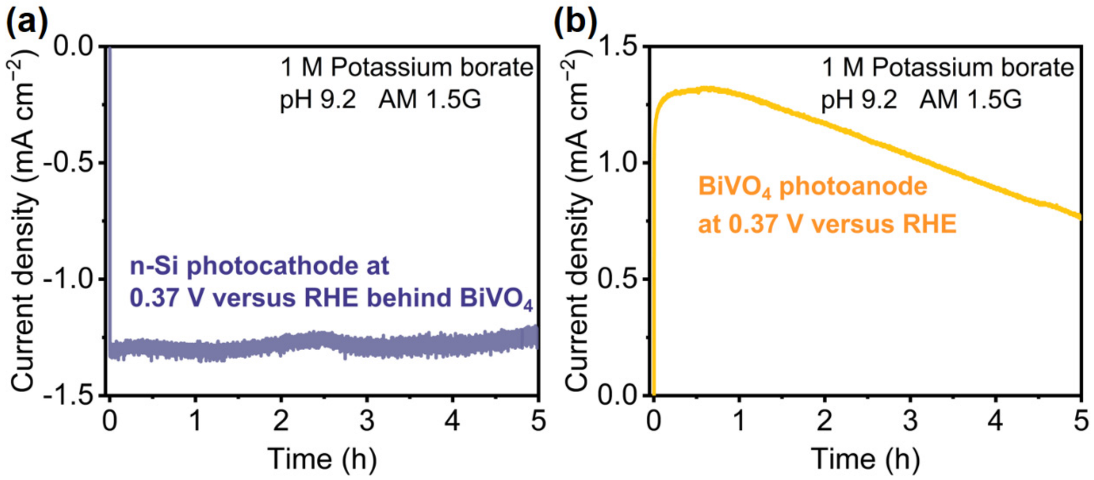

Supplementary Figure 15. Stability test of (a) n-Si MIS photocathode (behind $BiVO_4$) and (b) $BiVO_4$/FeOOH/NiOOH photoanode, at 0.37 V versus RHE with stirring under simulated AM 1.5G illumination. 1 M potassium borate buffer solution (pH 9.2) was used as an electrolyte.

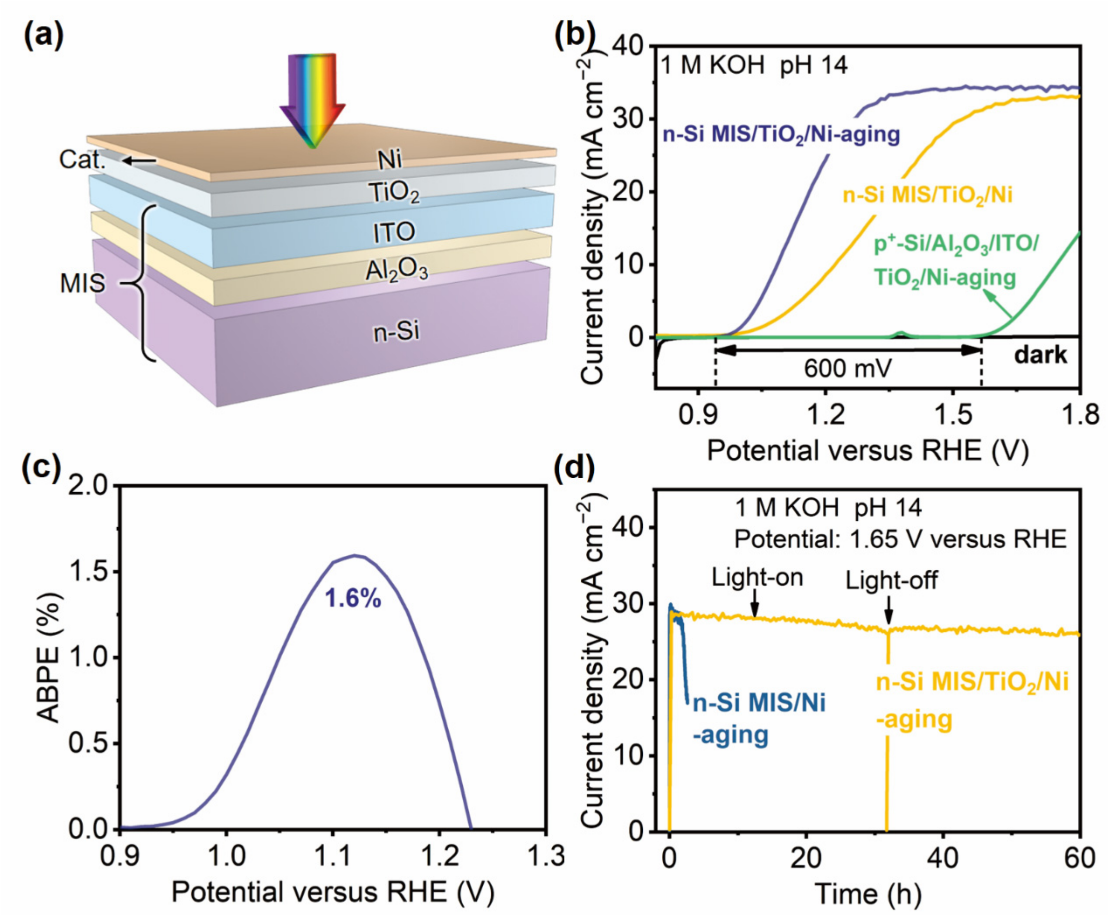

Supplementary Figure 16. PEC water oxidation performance of n-Si MIS photoanode. (a) The schematic of n-Si/Al_2O_3/ITO/TiO_2/Ni photoanode. (b) *J-V* curves of n-Si/Al_2O_3/ITO/TiO_2/Ni before and after aging and p^+-Si/Al_2O_3/ITO/TiO_2/Ni after aging. (c) ABPE curves of n-Si/Al_2O_3/ITO/TiO_2/Ni after aging. (d) Stability test of n-Si/Al_2O_3/ITO/Ni and n-Si/Al_2O_3/ITO/TiO_2/Ni photoanodes after aging at 1.65 V versus RHE in 1 M KOH electrolyte (pH 14) under simulated AM 1.5G illumination.

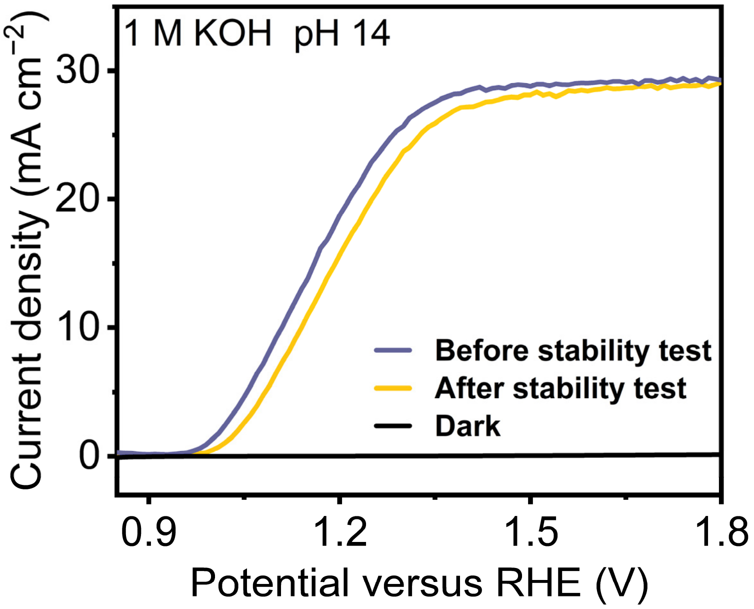

Supplementary Figure 17. *J-V* curves of n-Si MIS photoanodes before and after the 60 h stability test.

Supplementary Tables

Supplementary Table 1. Summary of recent representative reports on silicon-based photocathode for hydrogen evolution in Supplementary Fig. 6.

No.	Photocathode	Electrolyte	Reported stability (h)	J at 0 V_{RHE} (mA/cm^2)	V_{onset} (mV)	ABPE (%)	Ref.
1	p-Si/SiO_2/Pt/SiO_2	0.5 M H_2SO_4	12	30	242	0.3	*Nano Lett.* 2016 [1]
2	p-Si/$SrTiO_3$/Ti/Pt	0.5 M H_2SO_4	35	25.5	450	4.9	*Nat. Nanotechnol.* 2015 [2]
3	p-Si/SiO_x/Ti/Pt	0.5 M H_2SO_4	2	7.5	490	2.9	*Nat. Mater.* 2013 [3]
4	pn^+-Si/Ti/F:SnO_2/TiO_2/Ir	1 M KOH	24	35	574	10.9	*ACS Appl. Mater. Interfaces* 2014 [4]
5	pn^+-Si/Ti/TiO_2/Pt	1 M $HClO_4$	72	22	520	4.8	*J. Am. Chem. Soc.* 2013 [5]
6	pn^+-Si/Pt/TiO_2	1 M $HClO_4$	168	34.8	550	10.8	*J. Mater. Chem. A* 2017 [6]
7	pn^+-Si/Pt	0.5 M K_2SO_4 and H_2SO_4	2.5	28	410	8.9	*Appl. Phys. Lett.* 2016 [7]
8	n^+np^+-Si/Pt/TiO_2	1 M $HClO_4$	168	35	500	11.5	*Chem. Commun.* 2018 [8]
9	pn^+-Si/n^+-GaN/Pt	0.5 M H_2SO_4	113	38	500	10.5	*Nano Lett.* 2018 [9]
10	np^+-Si/Al_2O_3/Pt	0.5 M K_2SO_4 and H_2SO_4	73	30	520	8.68	*Appl. Phys. Lett.* 2015 [10]
11	ITO/Al_2O_3/n-Si/Ti/TiO_2/Pt	1 M $HClO_4$	316	35.2	570	10.3	This work

Supplementary Table 2. Summary of recent representative reports on silicon-based MIS photoanode for oxygen evolution.

No.	Photocathode	Electrolyte	Reported stability (h)	J at 1.23 V_{RHE} (mA/cm^2)	*V*onset (V *vs.* RHE)	ABPE (%)	Ref.
1	n-Si/SiO_x/Ni/NiO_x	1 M KOH	12	11.76	1.07	0.16	*Science* 2013 [11]
2	n-Si/SiO_x/Co/CoOOH	1 M KOH	2.5	16.4	1.05	0.54	*Nat. Mater.* 2015 [12]
3	n-Si/SiO_x,RCA/CoO_x/NiO_x	1 M KOH	1700	27.7	0.95	2.35	*Energy Environ. Sci.* 2015 [13]
4	n-Si/SiO_x/TiO_x/ITO/NiOOH	1 M LiOH	7	18	0.9	0.99	*J. Am. Chem. Soc.* 2016 [14]
5	n-Si/SiO_x,RCA/CoO_x	1 M KOH	2500	22.9	0.95	1.63	*Energy Environ. Sci.* 2016 [15]
6	n-Si/SiO_x/Al_2O_3/Pt/Ni	1 M KOH	200	19.2	0.95	0.98	*Nat. Commun.* 2017 [16]
7	n-Si/SiO_x/ Al_2O_3/Ni/NiO_x/NiOOH	1 M KOH	80	28	0.85	3	*Small Methods* 2019 [17]
8	n-Si/ZrO_2/NiFe	1 M KOH	100	26.6	0.96	-	*ACS Catal.* 2018 [18]
9	n-Si/SiO_x/NiFe	1 M NaOH	50	25.2	1.09	0.85	*Chem. Mater.* 2019 [19]
10	n-Si/TiO_2/NiAu	1 M KOH	20	18.8	1.03	0.78	*ACS Appl. Mater. Interfaces* 2018 [20]
11	n-Si/$NiSi_x$/NiO_x/Au	1 M NaOH	10	34	0.88	-	*ACS Appl. Energy Mater.* 2019 [21]
12	n-Si/Al_2O_3/ITO/TiO_2/Ni	1 M KOH	60	27.4	0.94	1.6	This work

Supplementary Experimental Procedures

Preparation of substrates.

Phosphorus-doped (n-type, (100)-oriented, double-side polished, resistivity 0.1-0.5 Ωcm, 500 μm), boron-doped (p-type, (100)-oriented, single-side polished, resistivity 1-10 Ωcm, 500 μm), degenerately boron-doped (p^+-type, (100)-oriented, single-side polished, resistivity 0.001-0.01 Ωcm, 500 μm) and degenerately arsenic doped (n^+-type, (100)-oriented, single-side polished, resistivity 0.001-0.005 Ωcm, 500 μm) Si wafers were purchased from MTI Corporation. All the Si wafers were first immersed in a piranha solution containing a mixture of sulfuric acid (H_2SO_4, 95.0-98.0%, Real & Lead Chemical Co., LTD.) and hydrogen peroxide (H_2O_2, 30% (w/w) in H_2O, contains stabilizer, Sigma-Aldrich) with a volume ratio of 3:1 to remove organic contaminants. Then the Si wafers were dipped into a 1% hydrofluoric acid (HF, ≥ 40.0%, Tianjin Fengchuan Chemical Reagent Technologies Co., LTD.) solution for 3 min to strip the native oxide, followed by rinsing with deionized water and drying with N_2. The native oxide thickness of the Si wafers was reduced to 0.6-0.8 nm after treatment as measured by spectroscopic ellipsometry (M-2000D, J. A. Woollam).

Fabrication of n-Si MIS photocathodes.

After cleaning by the piranha solution and HF solution, a 2.5 nm Al_2O_3 tunneling layer was deposited on the n-Si at 150 °C using a home-built atomic layer deposition (ALD) system with trimethyl aluminum (TMA, 99.999%, Suzhou Fornano Electronics Technology Co., LTD.) and H_2O (room temperature) as precursors. In a typical process, one ALD cycle consists of TMA dose for 0.04 s, N_2 purge for 5 s, H_2O dose for 0.3 s, and N_2 purge for 5 s. The growth rate of Al_2O_3 was 0.1 nm per cycle. Afterward, a 46 nm ITO (In_2O_3: SnO_2 = 90%: 10%, ≥ 99.99%) film was deposited using radio frequency (RF) sputtering to form a MIS junction. The Ar flow was kept at 20 sccm, and the working pressure was held at 0.2 Pa. The sputtering power was kept at 40 W and the deposition rate was approximately 3.833 Å s−1. The MIS junction was heated to 400°C for 30 min in a N_2 environment. Then, a 5.6 nm metallic Ti (≥ 99.99%) thin film was deposited on the other side of the Si wafer using DC sputtering to form an ohmic contact subsequently. The Ar flow was maintained at 20 sccm, the working pressure was 1 Pa and the sputtering power was 15 W. The deposition rate was approximately 0.933 Å s^{-1} under these conditions. After that, an 8 nm TiO_2 protect layer was deposited on the Ti film at 150°C using atomic layer deposition (ALD) with titanium (IV) i-propoxide (TTIP, 99.999%, Suzhou Fornano Electronics Technology Co., LTD., 70°C) and H_2O (room temperature) as precursors. One ALD cycle consists of a TTIP dose for 3 s, N_2 purge for 8 s, H_2O dose for 0.2 s, and N_2 purge for 8 s. The growth rate of TiO_2 was 0.027 nm per cycle. Finally, a 2 nm Pt (≥ 99.99%) layer as HER catalyst was deposited on the TiO_2 using DC sputtering to facilitate the surface reaction. The Ar flow was kept at 20 sccm, and the working pressure was 1 Pa. The sputtering power was 10 W and the deposition rate was approximately 2 Å s^{-1}. All the targets were purchased from Zhongnuo Advanced Material (Beijing) Technology Co., LTD. After depositions, Cu wire was connected to the ITO film by silver conductive adhesive (silver content 60%, resistivity 0.02 Ω/25 μm^2, Electron Microscopy China). The exposed edges and some parts of the front of the electrodes were sealed with an epoxy adhesive (DP420, 3M).

Fabrication of n-Si MIS photoanodes.

For the photoanodes, a 2.5 nm Al_2O_3 tunneling layer, a 20 nm ITO and an 8 nm TiO_2 protect layer were deposited on the n-Si in sequence under the same conditions as the photocathodes. Then, a 4 nm metallic Ni (≥ 99.99%) thin film was deposited on the TiO_2 film using DC sputtering. The Ar flow, working pressure, and sputtering power were kept at 20 sccm, 1 Pa, and 10 W, respectively. And the deposition rate was approximately 1 Å s^{-1}. The oxide layer on the backside of the Si wafer was removed with sandpaper, and then the residue was cleaned with ethanol. Next, a Ga-In alloy (75.5:24.5 wt%, ≥99.99% trace metals basis, Sigma-Aldrich) was rubbed on the Si to form an ohmic contact. The exposed edges and some parts of the front of the electrodes were sealed with an epoxy adhesive (DP420, 3M).

Fabrication of p-Si MIS photocathodes.

For the p-Si MIS photocathodes, a 2.5 nm Al_2O_3 tunneling layer, a 5.6 nm Ti, an 8 nm TiO_2 protect layer and 2 nm Pt were deposited on the p-Si in sequence under the same conditions as the photocathodes. The oxide layer on the backside of the Si wafer was removed with sandpaper, and then the residue was cleaned with ethanol. Next, a Ga-In alloy (75.5:24.5 wt%, ≥99.99% trace metals basis, Sigma-Aldrich) was rubbed on the Si to form an ohmic contact. The exposed edges and some parts of the front of the electrodes were sealed with an epoxy adhesive (DP420, 3M).

Fabrication of $BiVO_4$ photoanodes.

The $BiVO_4$ film was fabricated on FTO (F:SnO_2, 14 Ω per square, Wuhan Jinge-solar Energy Technology Co. LTD.) substrates by metalorganic decomposition method as previously reported [22]. In short, 1 M precursor solution was obtained by mixing 0.2425 g bismuth nitrate pentahydrate ($Bi(NO_3)_3 \cdot 5H_2O$, VR, Shanghai Aladdin Bio-Chem Technology Co. LTD.) and 0.1325 g vanadyl acetylacetonate ($VO(acac)_2$, 99.0%, J&K Scientific LTD.) in 500 µL dimethyl sulfoxide (DMSO, AR, Sigma-Aldrich.) and sonicated for 30 min at room temperature. Then the precursor solution was deposited onto preheated FTO (60°C) by spin coating at 1000 rpm for 20 s followed by 3000 rpm for 40 s, calculated in a tube furnace at 500°C for 2 h in the air. The obtained electrodes are soaked in 1 M KOH for 5 min to remove the excess VO_x species [23]. The obtained film was immersed in 1 M KBi buffer solution containing 0.2 M Na_2SO_3 (98%, Tianjin Guang Fu Fine Chemical Research Institute.) under simulated AM 1.5 G illumination for 10 min. For the catalyst, FeOOH layer is photoelectrodeposited on $BiVO_4$ in 0.1 M Ferrous sulfate ($FeSO_4 \cdot 7H_2O$, AR, Tianjin Kemiou Chemical Reagent Co. LTD.) solution at 0.25 V versus Ag/AgCl (total charge 45 mC cm^{-2}). Followed by the photoelectrodeposition of NiOOH layer in 0.1 M Nickel sulfate ($NiSO_4 \cdot 6H_2O$, 98.5%, Tianjin Kemiou Chemical Reagent Co. LTD.) solution at 0.11 V versus Ag/AgCl (total charge 22 mC cm^{-2}) and the electrodeposition of NiOOH in 0.1 M solution at 1.2 V versus Ag/AgCl for 1 min.

Photoelectrochemical measurements.

Using a three-electrode configuration, all PEC measurements were performed in electrolyte solutions. For photocathodes, the prepared electrode, a saturated Ag/AgCl electrode, and a platinum foil (2 × 2 cm^2) were used as a working electrode, a reference electrode, and a counter electrode, respectively. 1 M Perchloric acid ($HClO_4$, 70.0-72.0%, Tianjin Zhengcheng chemical

Co., LTD., pH 0) was used as the working electrolyte. The measured potentials versus SCE were converted to the RHE scale, $E_{RHE} = E_{SCE} + 0.059pH + 0.197$ [24]. For photoanodes, the prepared electrode, a Hg/HgO electrode and a platinum foil (2×2 cm^2) were used as a working electrode, a reference electrode, and a counter electrode, respectively. 1 M potassium hydroxide pellets (KOH, 99%, Tianjin Kemiou Chemical Reagent Co., LTD., pH 14) was used as the working electrolyte. Before test, the photoanode was immersed in 1M KOH electrolyte for 3 h-aging to oxide the thin Ni (4 nm) to the more transparent NiO_x or $Ni(OH)_2$ with higher catalytic activity [25]. The measured potentials versus Hg/HgO were converted to the RHE scale, $E_{RHE} = E_{Hg/HgO} + 0.059pH + 0.098$ [25]. *J-V* curves and chronoamperometry were measured by an electrochemical workstation (CompactStat.e20250, IVIUM) under the irradiation provided by an AM 1.5G solar simulator (PLS-SXE300D/300DUV, Beijing Perfectlight), and the light intensity was adjusted to 100 mW cm^{-2} against a calibrated Si photodiode (Thorlabs, Inc.). The *J-V* curves were measured at 50 mV s^{-1}. The active areas of the working electrode were determined by the software Image J.

The applied bias photon-to-current efficiency (ABPE) of the electrodes above was calculated from the *J-V* curves, according to the equation $ABPE = I\times \{(1.23 - |V_b|)/P\} \times 100\%$ [2]. Where I is the photocurrent density (mA cm^{-2}), V_b is the potential versus ideal counter electrode (V), and P is the incident illumination intensity (100 mW cm^{-2} in this work).

H_2 was collected and analyzed by an on-line gas chromatograph (7890B, Agilent Technologies) with a thermal conductivity detector (TCD) using N_2 as the carrier gas. The Faradaic efficiency (FE) for the H_2 product was calculated according to the equation FE (%) = (moles products × number of electrons needed)/ (moles of electrons passed) × 100%

The sustainable water reduction performance of the MIS photocathode and the sustainable water oxidation performance of the MIS photoanode were conducted at a constant potential of 0 V versus RHE and 1.65 V versus RHE, respectively. During the measurements, the electrolytes were continuously stirred using a magnetic stir bar.

Characterization.

The morphology was characterized using a field emission scanning electron microscope (FE-SEM, Hitachi S-4800, 5 kV). The thicknesses of the ITO, Al_2O_3, TiO_2, Pt and Ni layers on the polished Si (100) monitor substrate were obtained using a spectroscopic ellipsometer (M-2000 D, J. A. Woollam Co., Inc.) at incident angles of 60° and 70°, by fitting the amplitude ratio (Ψ) and phase shift (Δ) of polarized light with the Cauchy dispersion model for ITO, Al_2O_3 and TiO_2, and tabulated models for Pt and Ni. The Si monitor substrate was placed 5 mm next to the Si sample in the same batch. *J-V* curves of the solid-state cells were measured on a source-meter (Model 2450, Keithley Instruments) in the voltage range from −0.5 to 1.0 V. A Ga-In alloy was rubbed on the Si to make a back contact on the electrodes and Cu wire was connected to the metallic film by silver conductive adhesive. The transmission and reflection spectra were recorded in the range of 300-800 nm at room temperature by a SHIMADZU UV-2550 spectrophotometer.

Supplementary References

1. Labrador NY, Li X and Liu Y *et al.* Enhanced performance of Si MIS photocathodes containing oxide-coated nanoparticle electrocatalysts. *Nano Lett* 2016; **16**: 6452-9.
2. Ji L, McDaniel MD and Wang S *et al.* A silicon-based photocathode for water reduction with an epitaxial $SrTiO_3$ protection layer and a nanostructured catalyst. *Nat Nanotechnol* 2015; **10**: 84-90.
3. Esposito DV, Levin I and Moffat TP *et al.* H_2 evolution at Si-based metal-insulator-semiconductor photoelectrodes enhanced by inversion channel charge collection and H spillover. *Nat Mater* 2013; **12**: 562-8.
4. Kast MG, Enman LJ and Gurnon NJ *et al.* Solution-deposited F:SnO_2/TiO_2 as a base-stable protective layer and antireflective coating for microtextured buried-junction H_2-evolving Si photocathodes. *ACS Appl Mater Interfaces* 2014; **6**: 22830-7.
5. Seger B, Pedersen T and Laursen AB *et al.* Using TiO_2 as a conductive protective layer for photocathodic H_2 evolution. *J Am Chem Soc* 2013; **135**: 1057-64.
6. Fan R, Dong W and Fang L *et al.* More than 10% efficiency and one-week stability of Si photocathodes for water splitting by manipulating the loading of the Pt catalyst and TiO_2 protective layer. *J Mater Chem A* 2017; **5**: 18744-51.
7. Fan R, Tang C and Xin Y *et al.* Surface passivation and protection of Pt loaded multicrystalline pn^+ silicon photocathodes by atmospheric plasma oxidation for improved solar water splitting. *Appl Phys Lett* 2016; **109**: 233901.
8. Yin Z, Fan R and Huang G *et al.* 11.5% efficiency of TiO_2 protected and Pt catalyzed n^+np^+-Si photocathodes for photoelectrochemical water splitting: manipulating the Pt distribution and Pt/Si contact. *Chem Commun* 2018; **54**: 543-6.
9. Vanka S, Arca E and Cheng S *et al.* High efficiency Si photocathode protected by multifunctional GaN nanostructures. *Nano Lett* 2018; **18**: 6530-7.
10. Fan R, Min J and Li Y *et al.* n-type silicon photocathodes with Al-doped rear p^+ emitter and Al_2O_3-coated front surface for efficient and stable H_2 production. *Appl Phys Lett* 2015; **106**: 213901.
11. Kenney MJ, Gong M and Li Y *et al.* High-performance silicon photoanodes passivated with ultrathin nickel films for water oxidation. *Science* 2013; **342**: 836-40.
12. Hill JC, Landers AT and Switzer JA. An electrodeposited inhomogeneous metal-insulator-semiconductor junction for efficient photoelectrochemical water oxidation. *Nat Mater* 2015; **14**: 1150-5.
13. Zhou X, Liu R and Sun K *et al.* Interface engineering of the photoelectrochemical performance of Ni-oxide-coated n-Si photoanodes by atomic-layer deposition of ultrathin films of cobalt oxide. *Energy Environ Sci* 2015; **8**: 2644-9.
14. Yao T, Chen R and Li J *et al.* Manipulating the interfacial energetics of n-type silicon photoanode for efficient water oxidation. *J Am Chem Soc* 2016; **138**: 13664-72.
15. Zhou X, Liu R and Sun K *et al.* 570 mV photovoltage, stabilized n-Si/CoO_x heterojunction photoanodes fabricated using atomic layer deposition. *Energy Environ Sci* 2016; **9**: 892-7.
16. Digdaya IA, Adhyaksa GWP and Trzesniewski BJ *et al.* Interfacial engineering of metal-insulator-semiconductor junctions for efficient and stable photoelectrochemical water oxidation. *Nat Commun* 2017; **8**: 15968.

17. Luo Z, Liu B and Li H *et al.* Multifunctional nickel film protected n-Type silicon photoanode with high photovoltage for efficient and stable oxygen evolution reaction. *Small Methods* 2019; **3**: 1900212.
18. Cai Q, Hong W and Jian C *et al.* Insulator layer engineering toward stable Si photoanode for efficient water oxidation. *ACS Catal* 2018; **8**: 9238-44.
19. Li C, Huang M and Zhong Y *et al.* Highly efficient NiFe nanoparticle decorated Si photoanode for photoelectrochemical water oxidation. *Chem Mater* 2018; **31**: 171-8.
20. Hong W, Cai Q and Ban R *et al.* High-performance silicon photoanode enhanced by gold nanoparticles for efficient water oxidation. *ACS Appl Mater Interfaces* 2018; **10**: 6262-8.
21. Ying Z, Yang X and Tong R *et al.* Enhancing the efficiency and stability of NiO_x-based silicon photoanode via interfacial engineering. *ACS Appl Energy Mater* 2019; **2**: 6883-90.
22. Feng S, Wang T and Liu B *et al.* Enriched surface oxygen vacancies of photoanodes by photoetching with enhanced charge separation. *Angew Chem Int Ed* 2020; **59**: 2044-8.
23. Chang X, Wang T and Zhang P *et al.* Enhanced surface reaction kinetics and charge separation of p-n heterojunction $Co_3O_4/BiVO_4$ photoanodes. *J Am Chem Soc* 2015; **137**: 8356-9.
24. Zheng J, Lyu Y and Wang R *et al.* Crystalline TiO_2 protective layer with graded oxygen defects for efficient and stable silicon-based photocathode. *Nat Commun* 2018; **9**: 3572.
25. Digdaya IA, Trzesniewski BJ and Adhyaksa GWP *et al.* General considerations for improving photovoltage in metal-insulator-semiconductor photoanodes. *J Phys Chem C* 2018; **122**: 5462-71.
